# Regulation of Toxin Production in *Clostridium perfringens*

**DOI:** 10.3390/toxins8070207

**Published:** 2016-07-05

**Authors:** Kaori Ohtani, Tohru Shimizu

**Affiliations:** 1 Miyarisan Pharmaceutical Co., Ltd., 1-10-3, Kaminakazato, Kita-ku, Tokyo 114-0016, Japan; 2 Department of Bacteriology, Graduate School of Medical Science, Kanazawa University, 13-1 Takara-machi, Kanazawa, Ishikawa 920-8640, Japan

**Keywords:** *C. perfringens*, regulatory network

## Abstract

The Gram-positive anaerobic bacterium *Clostridium perfringens* is widely distributed in nature, especially in soil and the gastrointestinal tracts of humans and animals. *C. perfringens* causes gas gangrene and food poisoning, and it produces extracellular enzymes and toxins that are thought to act synergistically and contribute to its pathogenesis. A complicated regulatory network of toxin genes has been reported that includes a two-component system for regulatory RNA and cell-cell communication. It is necessary to clarify the global regulatory system of these genes in order to understand and treat the virulence of *C. perfringens*. We summarize the existing knowledge about the regulatory mechanisms here.

## 1. Introduction

*Clostridium perfringens* is a Gram-positive anaerobic spore-forming bacterium that is widely distributed in nature, especially in soil and the intestinal tracts of humans and animals. It causes clostridial myonecrosis (gas gangrene) and mild enterotoxemia in humans. *C. perfringens* is divided into five types (types A through E) (Table 1) depending on its major toxin (α-, β-, ε-, and ι-toxins) production [1,2,3]. The α-toxin (CPA) is conserved in all types of *C. perfringens* and the structural gene is located on the chromosome. Three other major toxins are encoded on the plasmid, and the classification of *C. perfringens* is based on the presence of plasmids encoding β-toxin (CPB, *cpb* gene), ε-toxin (ETX, *etx* gene), and ι-toxin (ITX, *iap* and *ibp* gene) [4]. Among the five types of *C. perfringens*, the type A strains are recognized as major pathogens in humans [3].

*C. perfringens* produces a wide variety of enzymes and toxins. A genome analysis showed that *C. perfringens* cannot synthesize many types of amino acids because it lacks many genes related to amino acid biosynthesis [5]. The growth of *C. perfringens* in a host organism, therefore, requires both the degradation of host tissues in small-size nutrients and the rapid transport of the nutrients into bacterial cells. This ability of *C. perfringens* is essential for it to survive and grow in the host. Once *C. perfringens* starts growing, the host organism imports sugar compounds from host tissues that are degrading, and *C. perfringens* then uses the sugar to produce energy through an anaerobic glycolysis pathway. During this process, *C. perfringens* produces abundant gas and makes the conditions more suitable for further growth.

*C. perfringens* produces various toxins, and the production of toxins is tightly regulated by specific gene regulatory systems [6]. For instance, the production of Clostridium perfringens enterotoxin (CPE) by a *C. perfringens* type A strain occurs only when the bacterium sporulates. In addition, many toxin genes are regulated by the VirS/VirR two-component system and an accessory gene regulator (*agr*) quorum-sensing (QS) system. Several two-component systems and RNA regulators comprise a tight network to control the timing of the toxin production.

In this paper, we summarize the progress of the research related to the regulation of toxin production by *C. perfringens*.

## 2. The Regulation of Toxin Genes by Two-Component Regulatory Systems

Two-component systems (TCSs) are important regulatory systems that sense the composition of the environment and transmit the information into the cell [7]. Normally, a TCS consists of the membrane-bound sensor histidine kinase (which senses the environment or stimuli) and a cytoplasmic response regulator that acts as a transcriptional regulator. The sensor histidine kinase transfers a phosphate group to the response regulator and activates the response regulator. The activated response regulator regulates the expression of many genes [8].

There are 48 genes for two-component regulatory systems in the *C. perfringens* strain 13 genome [5]. Twenty-eight of these genes are sensor histidine kinase genes, and the other 20 genes are response regulators. Several sensor histidine kinases and response regulators are orphans, and there are no response regulators or histidine kinase genes next to them [5]. Several TCSs in *C. perfringens* have been extensively analyzed, and here we discuss three TCSs related to toxin gene regulation.

### 2.1. The Regulation of Toxin Genes by the VirS/VirR System

One of the most important and well-characterized TCSs in *C. perfringens* is the VirR/VirS system. The VirR/VirS system was originally identified in 1994 as a regulator for the α-toxin gene *plc*, the κ-toxin gene *colA* and the θ-toxin gene *pfoA* [9,10]. The VirS/VirR system consists of a gene for the response regulator *virR* and a gene for the sensor histidine kinase *virS*. VirS has a relatively hydrophobic *N*-terminus with six transmembrane regions. The autophosphorylation site is located at a putative cytoplasmic loop between the *N*-terminal transmembrane 4 and 5. Three domains that are conserved in other sensor histidine kinases are located in the *C*-terminal domain. The *N*-terminus of VirR has a conserved domain that needs to receive a phosphate group from cognate sensor histidine kinase [9,11].

Three toxin genes, *plc*, *pfoA*, and *colA*, are regulated by the VirS/VirR system, and the modes of their regulation are different. The *pfoA* gene has major and minor promoters, and only the major promoter is dependent on the VirS/VirR system. The, *colA* gene has two major promoters, and only one of them is VirR/VirS-dependent. *plc* has only one promoter, and it is dependent on the VirR/VirS system [10].

There is no common motif for VirR binding in the promoter region of these three toxin genes, and it is thus suspected that there is a secondary regulator under the VirS/VirR system that acts to regulate these genes. To identify novel genes that are regulated by the VirS/VirR system, a differential display method has been used. The analysis revealed *vrr* gene (which encodes VR-RNA, VirR regulated RNA), and a comparison of the promoter regions of *pfoA* and *vrr* showed a VirR-binding motif (CCAnTT(*n* = 15)CCAGTT(*n* = 3)Cac) [12,13].

An analysis of the complete genome sequence of *C. perfringens* strain 13 [5] showed that there are five genes that have a VirR-binding motif in their promoter region. These five genes are *pfoA* (Perfringolysin O or theta toxin gene), *hyp7* (subsequently named *vrr*), *virT* (encoding a hypothetical protein gene), *virU* (encoding a hypothetical protein gene), and *ccp* (encoding alpha-clostripain) gene, and the expression of these five genes was suggested to be regulated by the VirS/VirR system [5]. VR-RNA, which is encoded by *vrr*, and VirT and VirU, which are encoded by *virT* and *virU*, respectively, have regulatory activity. These regulatory mechanisms are reviewed below in a later section. In a different strain, EHE-NE18, the VirR-binding motif was also identified in the promoter region of *netB*, a gene for the pore-forming toxin NetB [14]. NetB production is positively regulated by the VirR/VirS system in *C. perfringens* strain EHE-NE18 [14]. The binding motif was experimentally confirmed [13,15], and it was shown that the genes are regulated by the VirS/VirR system at the transcriptional level.

The VirS/VirR system is located on the chromosome, but it can regulate genes on both the chromosome and the plasmid. For example, collagen-adhesin gene (*cna*) and beta-2 toxin (*cpb2*), which are on plasmids in *C. perfringens* type A strain 13, are regulated by the VirS/VirR system. However, *cna* is negatively regulated, whereas *cpb2* is positively regulated by this system [16]. *C. perfringens* strains are classified into types A–E depending on the production of major toxins. Except for α-toxin gene, the genes of the major *C. perfringens* toxins are located on the plasmid.

*cpb* encodes *C. perfringens* beta toxin (CPB), which is a major toxin produced by type B and type C strains that contribute to hemorrhagic necrotic enteritis. The production of CPB by a type C strain has been reported to be regulated by the VirS/VirR system [17]. CPB is required for the intestinal virulence of type C strains [18]. VirS/VirR contributes to the pathogenicity of type C strains through the regulation of CPB production [17]. These data indicated that the VirS/VirR system is a key regulator to control the genes on both the chromosome and the plasmid.

It was reported that the VirS/VirR system is also important for sensing cells [19]: when a type C strain of *C. perfringens* came into contact with Caco-2 cells, the toxin production was quickly up-regulated, whereas a VirR mutant could not induce the toxin production even when the strain came into contact with the same type of cells. These data indicated that the VirS/VirR system is important for sensing the environment (especially cells in the environment) and for up-regulating the toxin production [19].

*C. perfringens* lacks many genes related to amino acid biosynthesis and, thus, its ability to distinguish different aspects of the environment and to upregulate the production of toxins and enzymes is important to its growth. The upregulation of toxin production by contact with Caco-2 cells thus appears to be one of the responses when *C. perfringens* recognizes the host cells and prepares to degrade the host cells [20]. However, it is not yet known how *C. perfringens* recognizes Caco-2 cells.

The regulation of toxins by the VirS/VirR system is not the regulation of only their expression. Iota toxin is the major toxin produced by *C. perfringens* type E strains. Iota toxin is produced as an inactive form, and proteolysis by a protease is needed to activate this toxin. It was reported that the cleavage of immature protein is accomplished by a VirS/VirR-dependent protease [21]. These data indicated that the activity of the iota toxin is regulated by the VirS/VirR system through the regulation of the protease activity required for the proteolysis [21].

In addition, as discussed below, this system has a secondary regulator, VR-RNA. Through VR-RNA, the VirS/VirR-VR-RNA cascade controls the expression of various gene categories [22]. The VirS/VirR system is, thus, a global gene regulator and is one of the most important regulators in *C. perfringens*.

### 2.2. The RevR System

The well-characterized secondary TCS in *C. perfringens* is the RevR system. RevR is similar to the response regulators PhoB and YycF of other Gram-positive bacteria [23]. PhoB is a response regulator of a TCS of *C. kluyveri* [24] and YycF is a response regulator of *Bacillus subtilis* [25]. RevR is a putative orphan response regulator, and there is no histidine kinase gene upstream or downstream of the *revR* gene. In other bacteria, *phoB* and *yycf* have cognate histidine kinases. A homology search for those histidine kinases showed that CPE1757 is a candidate for the cognate *revR* histidine kinase [26]. However, the cognate histidine kinase for *revR* has not been analyzed.

RevR appears to be a classical response regulator with an *N*-terminal receiver domain and a *C*-terminal domain with a putative winged helix-turn-helix motif. RevR has a regulatory effect on virulence-related genes in a VirS/VirR-independent manner [26]. A microarray analysis showed that more than 100 genes, including virulence-related genes and VirS/VirR-regulated genes, are regulated by RevR [26]. Among the virulence-related genes, the hyaluronidase genes *nagH* and *nagL* are regulated positively, and the sialidase gene *nanI* and the α-clostripain gene *ccp* are regulated negatively by this system [26]. Importantly, a RevR mutant strain showed attenuated virulence compared to the wild-type strain in a mouse myonecrosis model [26]. These data showed that RevR is important for the pathogenesis of *C. perfringens*.

Both the VirS/VirR and RevR systems are important in the regulation of virulence in *C. perfringens*.

### 2.3. ReeS (Regulator of Extracellular Enzymes Sensor)

An orphan histidine kinase called ReeS (regulator of extracellular enzymes sensor) was identified and extensively analyzed [23]. At the amino acid sequence level, ReeS retains conserved sensor histidine kinase domains, and also putative RE and YYY domains, which are conserved in hybrid sensor kinases. However, there is no potential DNA binding motif in the protein. There is no gene related to a two-component signal transduction system in close proximity to ReeS. It is, thus, thought that ReeS works as an orphan histidine kinase.

ReeS positively regulates the transcription of sialidase genes including the major sialidase gene, *nanI*; the minor sialidase genes *nanJ*. *nanI* and *nanJ* are also regulated by the VirS/VirR system, as discussed above [22]. However, the gene regulation by ReeS is independent from the VirS/VirR system. ReeS does not affect the gene expression of *pfoA*, *plc*, or *colA*, which are regulated by the VirS/VirR system, as well as that of the hyaluronidase genes *nagH* and *nagL*, which are regulated by RevR. It has been reported that sialidase is important for the virulence of other bacteria, but animal experiments using a ReeS mutant of *C. perfringens* showed that ReeS did not affect the virulence even when the sialidase production in the ReeS mutant was reduced [23].

TCS systems that have been analyzed showed that the sialidase gene *nanI* is regulated by all of the TCS systems that have been identified to date, i.e., the VirS/VirR, RevR, and ReeS systems [22,23,26]. The production of sialidase and sialic acid degradation are, thus, likely to be necessary for *C. perfringens* to acquire nutrients and survive in the host.

**Figure 1:**
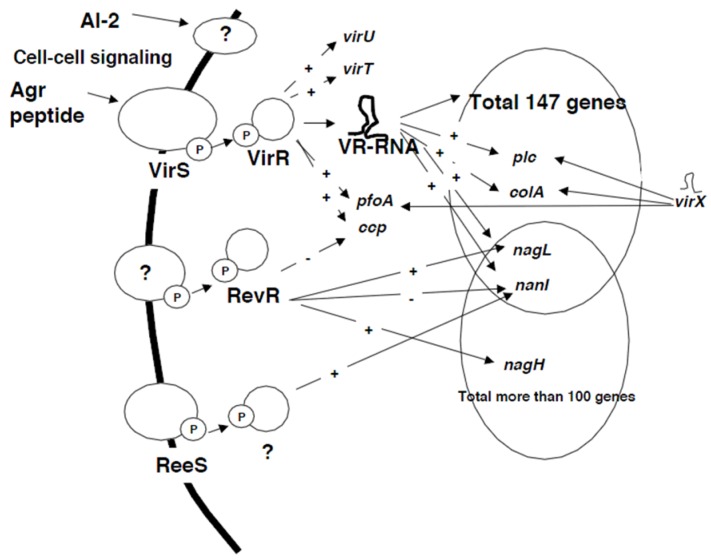
The regulatory networks of *C. perfringens* type A strain 13. VirS/VirR-VR-RNA cascade regulates total of 147 genes including toxin genes. The response regulator RevR and sensor histidine kinase ReeS also regulate several toxin genes. These two-component regulatory systems develop a complex toxin gene regulatory network. +: positive regulation, −: negative regulation.

These TCSs seem to comprise a complex regulatory network of toxin genes (Figure 1). The identification of cognate histidine kinases or response regulators of the orphan TCSs and analyses of other putative TCSs are expected.

### 2.4. Regulation by RNA Molecules

Many small RNA molecules with regulatory functions have been reported in both prokaryotes and eukaryotes [27]. Indeed, genome analyses showed that >10% of RNA coding regions have a role in gene regulation; these regulatory RNAs regulate transcription and/or translation by binding the target mRNAs or proteins [28]. It was reported that small RNAs in several pathogenic bacteria regulate virulence-related genes [27,29,30]. In *C. perfringens* there are RNA molecules that act as transcriptional regulators. The mechanisms underlying the regulation of toxin genes by RNA molecules are discussed in this section.

### 2.5. VR-RNA

VR-RNA was the first RNA molecule reported to have regulatory function in Clostridia. This RNA is regulated by the VirS/VirR system, and it was thus named VR-RNA for “VirR regulated RNA” [12,13]. VR-RNA was originally identified in a screening for VirS/VirR-regulated genes, and it was first thought that the VirR-regulated gene was *hyp7* gene (CPE0957). The mutant strain of this region showed a reduced amount of *plc* and *colA* transcripts. Further analysis showed that the region that is responsible for the regulation of *plc* and *colA* was not a protein-coding region of *hyp7* and that the RNA, itself, has a regulatory activity; *hyp7* was, therefore, renamed *vrr*. The region that is responsible for the regulatory activity is located in the small 3′-end region of the VR-RNA. A computer analysis of VR-RNA 386 nt from the transcription start site to the probable terminator region showed that the predicted structure is a stem-and-loop structure and that the 5′ and 3′ ends were predicted to pair [13].

VR-RNA was originally discovered as a secondary regulator of *plc* and *colA* in the regulation cascade of the VirS/VirR system. To identify the VirS/VirR-VR-RNA-regulated genes, microarray experiments were performed. The transcriptome data showed that 147 genes (30 single genes and 21 putative operons) including virulence-related genes are regulated by the VirR/VirS-VR-RNA cascade [22]. The VR-RNA-regulated virulence-related genes were not only *plc* and *cola*, but also sialidase genes (*nanI* and *nanJ*) and a hyaluronidase gene, *nagL*. In addition to virulence-related genes, the genes that are closely related to bacterial survival in the host tissue, including enzymes, transporters, and related genes for energy metabolism, are regulated by the VirS/VirR-VR-RNA cascade. These data indicated that the VirS/VirR-VR-RNA system is a global regulatory system that is needed to coordinate multiple functions of genes so that the *C. perfringens* can multiply in the host which would, in turn, cause the typical symptoms of gas gangrene.

The mechanisms underlying the regulation by VR-RNA of 147 genes in total [22] are not yet known. It is likely that other factors, e.g., VR-RNA binding proteins or third regulatory factors, exist in this system.

### 2.6. *VirT* and *VirU*

A genome sequence analysis of the *C. perfringens* strain 13 genome revealed five genes that have a VirR-binding motif [5]. Three of these five genes were demonstrated to regulate toxin genes as RNA regulators. One of the three genes is VR-RNA, discussed above. The other two genes are *virT* and *virU*. *virT* is located upstream of *ccp* (alpha-clostripain gene), which also has a VirR-binding motif. *virT* and *virU* transcription are strongly and positively regulated by VirR/VirS [31]. An analysis of *virT* mutant strain TS190 showed that VirT has a negative effect on the expressions of *pfoA* and *ccp*. *pfoA* and *ccp* have VirR-binding motifs in their promoter region, but VirT does not have a regulatory effect on *vrr* or *virU*, which also have a VirR-binding motif [31].

VirU, another RNA regulator, also affects toxin gene expression. A *virU* mutant has not been constructed, but an over-expression experiment showed that VirU regulates *pfoA*, *vrr*, *ccp*, and *virT* positively. VirT does not regulate the expression of *plc* or *colA*, whereas VirU had a slight effect on the expressions of *colA* and *plc* [31].

The regulation of toxin genes by VirT and VirU is not as strong as the regulation by VR-RNA, but VirT and VirU may fine-tune the transcription of virulence-related genes [31]. The entire regulatory system mediated by VirT and VirU is not yet clear, and broad-scale analyses are needed to address this topic.

### 2.7. *VirX*

VirX is another important RNA regulator in *C. perfringens* strain 13. VirX is an RNA transcribed from the CPE0646 region. It regulates *plc*, *colA*, and *pfoA* transcription in a VirS/VirR-independent manner [32]. *virX* gene encodes a 51-amino acid polypeptide, but it was shown that the RNA itself of this region has a regulatory effect on toxin genes. VR-RNA does not regulate *pfoA* transcription, but VirX positively regulates *pfoA* transcription. Thereby, *pfoA* is positively regulated by VirX at the mid-exponential phase, whereas *colA* and *plc* are positively regulated in the late-exponential phase [32]. The regulation of toxin genes is not so strong, but it was demonstrated that VirX is a negative regulator of sporulation [33]. It was thus suggested that between control/balance sporulation and toxin production is needed.

Since the enterotoxin gene *cpe* is expressed only when *C. perfringens* sporulates, the regulation of *cpe* by VirX has been analyzed, and the results showed that VirX regulates *cpe* transcription negatively in the sporulating condition.

## 3. Regulation of Toxin Genes by Cell-Cell Communication

Cell-cell communication is an important procedure used by bacteria to share information and, thus, ”talk“ to each other. Quorum sensing is the process that controls cell-to-cell communication. Bacteria sense the cell density or concentration of a signal substance. Once the concentration of signal substance reaches a threshold, this triggers gene regulation [34,35]. Several studies reported that the virulence factors of pathogenic bacteria are regulated by a quorum-sensing system [36,37,38].

Several cell-cell communication systems have been reported in many bacteria. Cell-cell communication in Gram-positive bacteria is mediated by two types of systems. The first system uses a peptide as a signal molecule to stimulate gene expression. This system, called an *agr* system, has been well studied in *Staphylococcus aureus* [39]. The second system is called the *luxS* system, which was first identified in *Vibrio harveyi* [40]. The *luxS* system is common to Gram-positive and Gram-negative bacteria and, thus, this system has been thought to be a tool that bacteria can use to communicate among each other beyond their own species.

*Clostridium perfringens* has both an *agr* quorum sensing system and a *luxS* system. The role of cell-to-cell communication in toxin gene expression is discussed below.

### 3.1. The Autoinducer 2 (AI-2) System

In the *C. perfringens* strain 13 genome, there is a *luxS* gene that is responsible for the production of AI-2 (autoinducer 2), which is the signal substance of quorum-sensing in both Gram-positive and Gram-negative bacteria. The AI-2 system was first observed in *Vibrio harveyi* as a quorum-sensing signal to stimulate its luminescence [40]. The culture supernatant of *C. perfringens* strain 13 stimulated the luminescence of *V. harveyi* strain, BB170, but the *luxS* mutant of *C. perfringens* strain 13 could not stimulate the luminescence [41]. These results showed that the *luxS* gene is responsible for the production of a signal substance that stimulates the luminescence of *V. harveyi*, AI-2.

The role of the *luxS* gene in *C. perfringens* was examined, and the authors reported that *luxS* positively regulates *plc*, *colA* and *pfoA* at the transcriptional level [41]. The transcription of *plc* and *colA* was reduced at the early log-phase, but *pfoA* transcription was reduced at the early to late-log phase in the *luxS* mutant [41]. The *luxS* mutant showed a recovered *pfoA* transcription level with the addition of wild-type supernatant or the supernatant of *Escherichia coli* DH5α carrying the *luxS* gene of *C. perfringens*. These data clearly indicated that the *luxS* gene is responsible for the production of AI-2 and that AI-2 regulates the toxin gene expression in *C. perfringens*.

As noted above, the AI-2 system is a common system in Gram-positive and Gram-negative bacteria [42]. Almost one-half of all bacteria the genome sequence of which is present in the database have the *luxS* gene [43]. It has, thus, been speculated that AI-2 is needed by bacteria that have an AI-2 system in order to communicate with each other beyond the species.

The importance of normal flora has been revealed, and AI-2 might be an important factor for maintaining the balance of the normal flora and pathogenic bacteria. Moreover, because it has been reported that AI-2 regulates many virulence factors, *luxS* is considered a therapeutic target in infectious diseases [44]. Further detailed analyses can be expected to contribute new research data in this area.

### 3.2. The agr System

The *agr* (accessory gene regulator) system is a well-characterized cell-cell communication system in Gram-positive bacteria, especially in *S. aureus* [45,46]. It is an important system that regulates virulence genes in a quorum-sensing manner in *S. aureus* and many other Gram-positive pathogenic bacteria [47,48,49]. In *S. aureus*, autoinducer propeptide (AIP) is produced from the *agrD* gene, and then AgrB is required to modify the AgrD propeptide. There is a two-component system, *agrAC*, downstream of *agrBD*. AgrC is a sensor protein for AgrD peptide, and once the concentration of AIP reaches a threshold, AgrC is activated. The activated AgrC then activates its cognate response regulator AgrA. Subsequently, activated AgrA regulates the RNA regulator RNAIII located upstream of the *agr* operon.

RNAIII regulates the transcription of various virulence genes in *S. aureus*. A homologous gene of *S. aureus*, *agrBD* was identified in the *C. perfringens* strain 13 genome, and the amino acid sequence showed that there is a conserved cysteine residue, which is important for the formation of a thiolactone ring, in the AgrD of *C. perfringens.* However, there is no TCS upstream or downstream of *agrBD* in the *C. perfringens* strain 13 genome.

A mutant strain of *agrBD*, TS230, showed weak hemolysis on a blood agar plate [50]. However, TS230 recovered hemolysis when the strain was cross-streaked with TS133 (a VirSR mutant that is hemolysis-negative, but produces a signal substance) from just after the crossing point [50]. This finding indicates that strain TS230 lost the signal substance to stimulate θ-toxin (or PFO) production, but if TS230 receives the signal from another strain, it can recover the toxin production. There are several studies from the 1970s that concern toxin production by *C. perfringens* [51,52,53,54]. Those studies describe two types of strains that were θ-toxin-negative even though they had a θ-toxin gene. One type of these strains cannot recover the toxin production by culture with other toxin-negative strains, whereas the other type can recover the toxin production [51]. In light of those findings, it has been thought that there must be a signal substance to stimulate θ-toxin production in *C. perfringens*. It seems that the *agr* system is closely related to this phenomenon.

The mutant strain of *agrBD*, TS230, reduced the transcription of *plc*, *colA*, and *pfoA* and recovered the transcription by complementation of *agrBD* gene [50], indicating that the *agr* system in *C. perfringens* regulates at least *plc*, *colA*, and *pfoA* genes among the virulence-related genes. In another report using the non-foodborne human gastrointestinal disease strain F5603, it has been shown that *agrB* regulates the production of CPE and CPB2 positively [55]. Moreover, the supernatant of the wild-type strain or complemented strain can stimulate the transcription toxin genes in TS230 [50]. These data indicated that the *agrBD* region of *C. perfringens* is responsible for the production of a signal substance.

An experiment using a *agrBD-virSR* double-mutant strain showed that the supernatant of wild-type *C. perfringens* strain 13 could not stimulate the toxin gene expression [50], suggesting that VirS is one of the sensor proteins for the signal peptide. Although a genome analysis showed that there is no TCS system around the *agrBD* gene as discussed above, these data indicated that VirSR corresponds to AgrAC of *S. aureus*. In addition, in *S. aureus*, RNAIII is a key factor of the *agr* system and AgrAC regulates RNAIII transcription; RNAIII then regulates the expression of many genes [39]. The manner of gene regulation by RNAIII seems to correspond to that of the VR-RNA of *C. perfringens*. In *S. aureus*, regulatory genes are clustered in the genome, but in *C. perfringens*, *virS/virR*, VR-RNA and *agrBD* are scattered in the genome.

The signal peptide that stimulates gene expression is produced from *agrD*. In *S. aureus*, amino acid sequences of AgrD are classified into four groups depending on the amino acid sequences [56]. In contrast, there is no such sequence variation in the *C. perfringens* AgrD amino acid sequence.

In other toxin-type *C. perfringens* strains, an *agr* system has been reported to have an important role in toxin production (Table 2). The five *C. perfringens* types depend on the production of major toxins. The *agrB* null mutant showed less CPB production and recovered the production by complementation with *agrB* in *C. perfringens* type B strains CN1793 and CN1795; however, the productions of epsilon toxin (ETX) and CPB2 were not affected by *agrB* mutation [57]. Experiments with type C strain CN3685 also showed that CPB production is regulated by the *agr* system [58]. It was also shown that the *agr* system is required to cause necrotizing enteritis by *C. perfringens* type B strain CN3685 [58].

CPB is produced by type B and type C strains, and the above findings showed that the *agr* system could regulate the CPB production in both types of strains. In type D strain CN3718 too, the *agr* system has an important role in the strain’s virulence. Type D strains produce ETX, which is a pore-forming toxin considered the major virulence factor of type B and D strains. ETX production has been reported to be regulated by the *agr* system [59]. ETX production in a type B strain was not affected by the *agr* system, but in a type D strain it was affected by the *agr* system [59].

There are some differences in regulatory systems among the strains even when they have the same regulator and toxin genes [57,59]. Interestingly, the signal that is produced from *agrD* has been thought to be a signal for VirS. However, in the regulation of ETX production in type D strain CN3718, ETX production was regulated by the *agr* system, but not by the VirS/VirR system. This was a first report showing that the *agr* system does not always activate the VirS/VirR system [59].

Overall, the *agr* system has a crucial role in the virulence factor production and pathogenesis in *C. perfringens*.

## 4. Other Types of Regulation

### CodY

Several regulatory proteins that are common in Gram-positive bacteria and related to metabolism have been reported. CodY protein is one such protein. CodY is thought to be involved in the adaptive response of Gram-positive bacteria to their environment. CodY senses the nutrient conditions of bacteria by binding GTP or branched-chain amino acid (BCAA) in the cell [61]. When the amount of GTP or BCAA is sufficient, CodY binds to the promoter region of the regulated genes in complex with GTP or BCAA. In the stationary phase, there are less nutrients, and CodY is not in complex with GTP or BCAA and has decreased affinity for the binding region of target genes.

It was reported that in low G + C Gram-positive bacteria, CodY regulates several virulence-related genes [62,63]. For instance, the production of toxin A and toxin B are regulated by CodY in *C. difficile* [64]. The function of CodY in *C. perfringens* type D strain CN3718 was investigated, and the analysis of a CodY mutant showed that CodY did not affect the production of PFO (θ-toxin) or PLC (α-toxin), but it positively regulated the production of ETX (ε-toxin) [60]. In contrast, the production of sialidase was negatively affected by CodY, mainly by the regulation of NanH.

The regulation of ETX occurs by CodY protein binding to the promoter region of the *etx* gene. A gel mobility shift assay showed that the CodY binding box is located between 21 and 354 bp upstream of the *etx* start codon [60]. However, the relationship between GTP and CodY or the nutrient condition has not been identified. In other bacteria, it has been reported there is a palindromic binding motif in the promoter region of regulated genes, and approx. Five percent of the genes in the genome are regulated by CodY [65]. It is, thus, likely that in *C. perfringens*, too, CodY has a global regulatory function. Additional studies are needed to elucidate the regulatory system involving CodY.

## 5. Conclusions

There are complex toxin gene regulatory systems in *C. perfringens* (Figure 1). Here we focused on the regulatory network of toxin genes, but the same network, i.e., the VirS/VirR-VR-RNA cascade, regulates various categories of genes, including extracellular enzymes, transporters, and genes for energy metabolism. The VirS/VirR-VR-RNA cascade is a global regulator that may control multiple cellular functions that enable *C. perfringens* to survive and multiply in infectious conditions. The VirS/VirR-VR-cascade is just one example; other regulators also regulate multifunction genes.

It may be difficult for *C. perfringens* to obtain nutrients from its host under infectious conditions, but it must get the nutrients to survive and multiply by degrading host tissue. The networks that have been demonstrated to regulate toxin genes would be required to coordinate the expression of genes that are needed to degrade the host tissue into small-size nutrients. This process turns into the necrotic infection of *C. perfringens*.

Many regulatory systems of toxin genes have been identified, but compared with other pathogenic bacteria we still have limited knowledge about the regulation of virulence. In other pathogenic clostridia, e.g., *C. difficile* and *C. botulinum*, it has been reported that certain amino acids have an effect on toxin production. The repression of toxin production by a mixture of specific amino acids is mediated by CodY. The presence of glucose represses the toxin production through CcpA [66]. The importance of alternative sigma factors for the regulation of toxin production has also been reported in clostridia, especially in *C. botulinum* and *C. difficile* [66,67]. Similar mechanisms to control the toxin production might exist in *C. perfringens*. Compared with *C. difficile*, less is known about the regulation of toxins by metabolites or alternative sigma factors in *C. perfringens*. More extensive research on the regulatory mechanisms of virulence of *C. perfringens* is highly desired. The elucidation of the detailed regulatory network of toxin production could enable the development of molecular-based preventive and therapeutic techniques for combatting *C. perfringens* infections and infectious diseases overall.

## Figures and Tables

**Table 1:** Classification of *C. perfringens*.

Type	Toxins Produced			
	Alpha	Beta	Epsilon	Iota
A	+	−	−	−
B	+	+	+	−
C	+	+	−	−
D	+	−	+	−
E	+	−	−	+

**Table 2:** Regulation of toxin production or toxin gene expression in *C. perfringens*.

Type	Toxin (gene)	Regulation by VirR/VirS Ref: [9,10,14,16,17,22,57]	Regulation by agr Ref: [50,55,57,58,59]	VR-RNA Ref: [12,13,22]	VirT Ref: [31]	VirU Ref: [31]	VirX Ref: [32]	CodY Ref: [60]	ReeS Ref: [23]	RevR Ref: [26]
A	CPA (*plc*)	Yes (Yes)	Yes (Yes)	(Yes)	No	No	(Yes)	NR	No	No
	PFO (*pfoA*)	Yes (Yes)	Yes (Yes)	No	(Yes)	(Yes)	(Yes)	NR	No	No
	collagenase (*colA*)	(Yes)	(Yes)	(Yes)	No	No	(Yes)	NR	No	No
	sialidase (*nanI*)	Yes	NR	(Yes)	NR	NR	NR	NR	(Yes)	(Yes)
	Hyaluronidase (*nagH*)	(Yes)	NR	(Yes)	NR	NR	NR	NR	No	(Yes)
	Hyaluronidase (*nagL*)	(Yes)	NR	(Yes)	NR	NR	NR	NR	No	(Yes)
	α-clostripain (*ccp*)	(Yes)	NR	No	(Yes)	(Yes)	NR	NR	No	(Yes)
	CPE (*cpe*)	NR	Yes	NR	NR	NR	NR	NR	No	No
	NetB (*netB*)	Yes	NR	NR	NR	NR	NR	NR	No	No
	CPB2 (*cpb2*)	Yes	Yes	(Yes)	NR	NR	NR	NR	No	NR
B	CPA (*plc*)	NR	Yes	NR	NR	NR	NR	NR	NR	NR
	PFO (*pfoA*)	NR	Yes	NR	NR	NR	NR	NR	NR	NR
	CPB (*cpb*)	NR	Yes	NR	NR	NR	NR	NR	NR	NR
	ETX (*etx*)	NR	No	NR	NR	NR	NR	NR	NR	NR
	CPB2 (*cpb2*)	NR	No	NR	NR	NR	NR	NR	NR	NR
C	CPA (*plc*)	Yes	Yes	NR	NR	NR	NR	NR	NR	NR
	PFO (*pfoA*)	Yes	Yes	NR	NR	NR	NR	NR	NR	NR
	CPB (*cpb*)	Yes	Yes	NR	NR	NR	NR	NR	NR	NR
D	CPA (*plc*)	Yes	Yes	NR	NR	NR	NR	No	NR	NR
	PFO (*pfoA*)	Yes	Yes	NR	NR	NR	NR	No	NR	NR
	ETX (*etx*)	No	Yes(Yes)	NR	NR	NR	NR	Yes	NR	NR
E	ITX (*iap* and *ibp*)	NR (for activity Yes)	NR	NR	NR	NR	NR	NR	NR	NR

NR: not reported, Yes or No: regulation of toxin production level is reported, (Yes or No): regulation of gene expression is reported.
